# A vicious cycle between acid sensing and survival signaling in myeloma cells: acid-induced epigenetic alteration

**DOI:** 10.18632/oncotarget.11927

**Published:** 2016-09-10

**Authors:** Ryota Amachi, Masahiro Hiasa, Jumpei Teramachi, Takeshi Harada, Asuka Oda, Shingen Nakamura, Derek Hanson, Keiichiro Watanabe, Shiro Fujii, Hirokazu Miki, Kumiko Kagawa, Masami Iwasa, Itsuro Endo, Takeshi Kondo, Sumiko Yoshida, Ken-Ichi Aihara, Kiyoe Kurahashi, Yoshiaki Kuroda, Hideaki Horikawa, Eiji Tanaka, Toshio Matsumoto, Masahiro Abe

**Affiliations:** ^1^ Department of Hematology, Endocrinology and Metabolism, Tokushima University Graduate School, Tokushima, Japan; ^2^ Department of Orthodontics and Dentofacial Orthopedics, Tokushima University Graduate School, Tokushima, Japan; ^3^ Department of Biomaterials and Bioengineerings, Tokushima University Graduate School, Tokushima, Japan; ^4^ Department of Histology and Oral Histology, Tokushima University Graduate School, Tokushima, Japan; ^5^ Division of Transfusion Medicine and Cell Therapy, Tokushima University hospital, Tokushima, Japan; ^6^ Department of Hematology and Oncology, RIRBM, Hiroshima University, Hiroshima, Japan; ^7^ Support Center for Advanced Medical Sciences, The University of Tokushima Graduate School, Tokushima, Japan; ^8^ Fujii Memorial Institute for Medical Research Tokushima University Graduate School, Tokushima, Japan

**Keywords:** multiple myeloma, acidic microenvironment, HDAC, Sp1, DR4

## Abstract

Myeloma (MM) cells and osteoclasts are mutually interacted to enhance MM growth while creating acidic bone lesions. Here, we explored acid sensing of MM cells and its role in MM cell response to acidic conditions. Acidic conditions activated the PI3K-Akt signaling in MM cells while upregulating the pH sensor transient receptor potential cation channel subfamily V member 1 (TRPV1) in a manner inhibitable by PI3K inhibition. The acid-activated PI3K-Akt signaling facilitated the nuclear localization of the transcription factor Sp1 to trigger the expression of its target genes, including *TRPV1* and *HDAC1.* Consistently, histone deacetylation was enhanced in MM cells in acidic conditions, while repressing a wide variety of genes, including *DR4*. Indeed, acidic conditions deacetylated histone H3K9 in a *DR4* gene promoter and curtailed *DR4* expression in MM cells. However, inhibition of HDAC as well as either Sp1 or PI3K was able to restore *DR4* expression in MM cells suppressed in acidic conditions. These results collectively demonstrate that acid activates the TRPV1-PI3K-Akt-Sp1 signaling in MM cells while inducing HDAC-mediated gene repression, and suggest that a positive feedback loop between acid sensing and the PI3K-Akt signaling is formed in MM cells, leading to MM cell response to acidic bone lesions.

## INTRODUCTION

Multiple myeloma (MM) has a unique propensity to develop and expand almost exclusively in the bone marrow and generates destructive bone disease. MM cells enhance osteoclastogenesis [1–4] while suppressing osteoblastogenesis [5–9], leading to devastating bone destruction with rapid loss of bone. Along with the progression of bone disease, the bone marrow microenvironment is skewed by MM cells, which underlies the unique pathophysiology of MM and confers aggressiveness and drug resistance in MM cells [10, 11]. While MM cells perturb bone metabolism to develop bone destruction, a crosstalk between MM cells and the microenvironment in bone lesions leads to a progressive vicious cycle phase of tumor growth and bone destruction [11–13].

Cancer cells, including MM cells, produce large amounts of protons and lactate as a consequence of anaerobic glycolysis (the Warburg effect) as well as low O_2_ conditions, which leads to extracellular acidification (pH 6.2–6.8) in cancer lesions compared to pH 7.2–7.4 in normal tissues [14–18]. The extracellular pH of acid-producing osteoclasts (OCs) is generally accepted to be below pH 5.5 [19]. Therefore, MM-OC interactions in bone lesions in MM appear to create a highly acidic milieu due to abundant production of protons by OCs and lactate by proliferating glycolytic MM cells. As a result, MM cells need to respond to acidic tumor microenvironments to survive. Although tumor acidity has been shown to blunt the activity of immune effecter cells [16, 20–22]. The precise mechanisms underlying response of MM cells to acidic tumor conditions remain largely unknown.

Transient receptor potential cation channel subfamily V member 1 (TRPV1) is discovered as a receptor of the capsaicin and a family member of transient receptor potential ion channels promoting ion inflows, and excited by the vanilloid capsaicin, and also activated by noxious stimuli, including heat and extracellular acidification. We demonstrate herein that acidic conditions activate the PI3K-Akt survival signaling in MM cells to upregulate TRPV1 and thereby acid sensing. The activation of the PI3K-Akt pathway facilitated nuclear localization of Sp1, a transcription factor responsible for HDAC1 as well as TRPV1 expression in MM cells in acidic conditions. Consistently, histone H3 and histone H4 were deacetylated in MM cells in acidic conditions, while repressing a wide variety of genes, including *DR4*. Therefore, MM cells are suggested to sense acid more efficaciously in acidic conditions to activate survival signaling and respond to an acidic microenvironment created in MM bone lesions.

## RESULTS

### Activation of the PI3K-Akt pathway in MM cells by acid

A microenvironment in malignant tumors is generally accepted to be acidic. To clarify mechanisms by which MM cells survive against an acidic insult and respond to an acidic tumor microenvironment, we first measured actual pH values in MM subcutaneous tumors in mouse models. The average pH value was 6.86 inside tumors in 8 mice, while 7.35 outsides of the tumors. Therefore, we set pH6.8 as an acidic condition in the following experiments.

**Figure 1:**
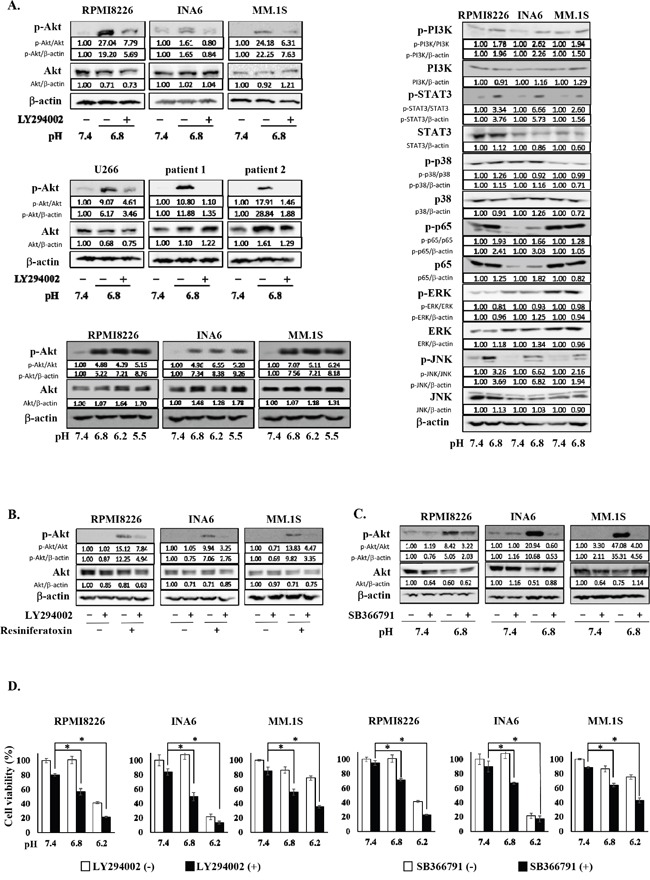
The activation of the PI3K-Akt pathway in MM cells under an acidic 8 condition **A.** RPMI8226, INA6, MM.1S, U266 MM cell lines, and primary MM cells isolated from patients with MM were cultured for 30 minutes at pH7.4 or pH6.8 with or without LY294002 at 10 μM as indicated (left, upper and middle). Lactic acid or sodium hydroxide was added to adjust pH of media to the indicated values. Cell lysates were extracted, and the protein levels of Akt and phosphorylated Akt were analyzed by Western blotting. RPMI8226, INA6, MM.1S MM cell lines were cultured for 30 minutes at pH7.4, pH6.8, pH6.2 or pH5.5. The protein levels of Akt and phosphorylated Akt were analyzed by Western blotting (left, lower). RPMI8226, INA6, MM.1S MM cell lines were cultured for 30 minutes at pH7.4 or pH6.8. The protein levels of PI3K, phosphorylated PI3K, STAT3, phosphorylated STAT3, p38, phosphorylated p38, p65, phosphorylated p65, ERK, phosphorylated ERK, JNK, and phosphorylated JNK were analyzed by Western blotting (right). β-actin was used as a protein loading control. **B.** RPMI8226, INA6, and MM.1S cells were cultured at pH7.4. The cells were treated for 30 minutes with the TRPV1 agonist resiniferatoxin at 1 μM or LY294002 at 10 μM alone or both in combination as indicated. The protein levels of Akt and phosphorylated Akt were analyzed by Western blotting. β-actin was used as a protein loading control. **C.** RPMI8226, INA6, and MM.1S cells were cultured for 30 minutes at pH7.4 or pH6.8 in the presence or absence of the TRPV1 antagonist SB366791 at 10 μM. The protein levels of Akt and phosphorylated Akt were analyzed by Western blotting. β-actin was used as a protein loading control. **D.** RPMI8226, INA6, and MM.1S cells were cultured in triplicate at pH7.4, pH6.8 or pH6.2 for 24 hours in the presence or absence of LY294002 at 10 μM or SB366791 at 10 μM. Cell viability was determined by a WST-8 assay. Results are expressed as % change from the baseline with the mean +/− SD. *, p <0.01.

Because the PI3K-Akt pathway is one of the most important survival signaling pathways in MM cells [23, 24, 25], we examined whether an acidic condition affects the PI3K-Akt signaling pathway. All MM cell lines as well as primary MM cells tested showed the phosphorylation of Akt when cultured under acidic conditions, which was abrogated by addition of the PI3K inhibitor LY294002 (Figure 1A). Phosphorylation of Akt was triggered at pH 6.8 or below but not at pH7.4. These results demonstrate that MM cells sense acid and activate the PI3K-Akt survival pathway in response to acid.

In addition, we looked at the activation of different signaling pathways in myeloma cells in an acidic condition, including PI3K-Akt, JAK-STAT3, NF-κB, ERK, p38MAPK and stress activated protein kinase-1 (JNK) pathways, and found the phosphorylation of STAT3, p65 and JNK aside from PI3K in MM cells cultured at pH6.8 (Figure 1A). Therefore, Jak2/STAT3, NF-κB and JNK pathways appear to be also activated in MM cells in acidic conditions.

### Acid sensing and activation of the PI3K-Akt pathway in MM cells via TRPV1

Extracellular pH sensors have been demonstrated to play an important role in sensing ambient acidity by tumor cells [26–29]. TRPV1 is among major pH sensors expressed in cancer cells [30–33], which belongs to an acid-sensitive ion channel. Treatment with the TRPV1 agonist resiniferatoxin potently induced the phosphorylation of Akt in MM cells, which was almost completely abrogated by addition of the PI3K inhibitor LY294002 (Figure 1B), indicating activation of the PI3K-Akt pathway in MM cells directly via TRPV1. Furthermore, the acid-induced phosphorylation of Akt in MM cells was mostly suppressed by addition of the TRPV1 antagonist SB366791 (Figure 1C), suggesting a predominant role of TRPV1 in acid-induced activation of the PI3K-Akt pathway in MM cells.

Although MM cell viability was marginally affected at pH6.8, MM cells underwent cell death at pH6.8 upon treatment with LY294002, and to a lesser extent with the TRPV1 antagonist SB366791 (Figure 1D). Although viability of the MM cells was reduced at pH6.2, LY294002 and SB366791 still increased cell death in RPMI8226 and MM.1S cells. INA6 cells were mostly dead only when cultured at pH6.2. These results suggest the critical role of TRPV1 in activation of the PI3K-Akt pathway in MM cells and their survival in response to acid.

### TRPV1 upregulation via the PI3K-Akt pathway in MM cells in an acidic condition

**Figure 2:**
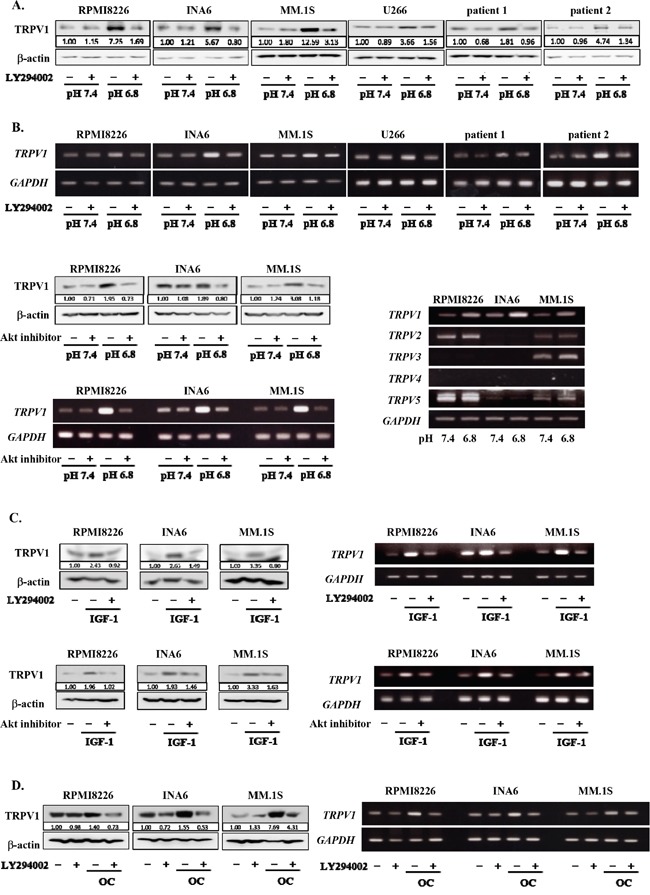
Up-regulation of TRPV1 expression in MM cells under an acidic condition RPMI8226, INA6, MM.1S, U266 MM cell lines as well as primary MM cells isolated from patients with MM were cultured at pH7.4 or pH6.8 with or without LY294002 at 10 μM as indicated. Lactic acid or sodium hydroxide was added to adjust pH of media to the indicated values. **A.** After culturing for 24 hours, the protein levels of TRPV1 were analyzed by Western blotting. **B.** After culturing for 6 hours, *TRPV1* mRNA expression was analyzed by RT-PCR (upper). RPMI8226, INA6, and MM.1S MM cell lines were cultured at pH7.4 or pH6.8 with or without Akt inhibitor VIII at 10 μM as indicated. After culturing for 24 hours, the protein levels of TRPV1 were analyzed by Western blotting. After culturing for 6 hours, *TRPV1* mRNA expression was analyzed by RT-PCR (lower, left). RPMI8226, INA6 and MM.1S MM cell lines were cultured at pH7.4 or pH6.8. After culturing for 6 hours, *TRPV1, TRPV2, TRPV3, TRPV4*, and *TRPV5* mRNA expression was analyzed by RT-PCR (lower, right). **C.** RPMI8226, INA6, and MM.1S cells were cultured at pH7.4 with or without rhIGF-1 at 10 nM. LY294002 or Akt inhibitor VIII was added at 10 μM as indicated. After culturing for 24 hours, the protein levels of TRPV1 were analyzed by Western blotting. After culturing for 12 hours, *TRPV1* mRNA expression was analyzed by RT-PCR. **D.** RPMI8226, INA6, and MM.1S cells were cultured at pH7.4 alone or in cocultures with osteoclasts generated from human peripheral blood monocytes as described in Materials and Methods. After culturing for 24 hours, the protein levels of TRPV1 were analyzed by Western blotting. After culturing for 6 hours, *TRPV1* mRNA expression was analyzed by RT-PCR. β-actin was used as a protein loading control. *GAPDH* was used as an internal control.

To further clarify the mechanism of acid sensing of MM cells, we next investigated the expression levels of TRPV1 in MM cells in acidic conditions. Of interest, the expression of TRPV1 was upregulated at protein as well as mRNA levels in all MM cell lines and primary MM cells tested at pH6.8 compared to those at pH7.4, which was abolished by addition of the PI3K inhibitor LY294002 as well as an Akt inhibitor (Figures 2A and 2B). Although TRPV1 was constitutively expressed in all MM cell lines tested and upregulated in an acidic condition, expression of TRPV2-5 was found to vary among the MM cells. These results suggest formation of positive feedback loop between acid sensing by TRPV1 and activation of the PI3K-Akt survival pathway.

IGF-1 activates the PI3K-Akt survival pathway as one of the most important survival factors for MM cells in the bone marrow [34, 35]. We previously reported that cocultures with acid-producing OCs also potently activate the PI3K-Akt survival pathway in MM cells [36]. To further confirm the role of the PI3K-Akt pathway in up-regulation of TRPV1 expression in MM cells, we therefore examined the effects of IGF-1 as well as OCs. Addition of rh IGF-1 or cocultures with OCs enhanced *TRPV1* mRNA expression in MM cells even at pH7.4 in a manner inhibitable by LY294002 (Figure 2C and Figure 2D, respectively). The IGF-1-induced upregulation of *TRPV1* mRNA expression was further confirmed to be abolished by the Akt inhibitor. These results collectively demonstrate the critical role of the PI3K-Akt pathway in upregulation of TRPV1 expression in MM cells.

### Acid-induced Sp1 nuclear localization and thereby TRPV1 upregulation in MM cells

**Figure 3:**
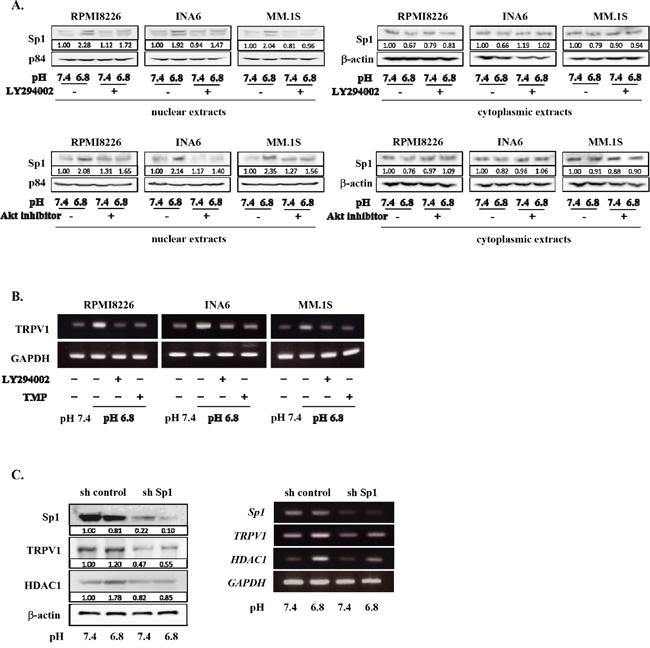
Acid-induced Sp1 nuclear localization and TRPV1 up-regulation in MM cells **A.** RPMI8226, INA6, and MM.1S cells were cultured for 24 hours at pH7.4 or pH6.8 with or without LY294002 or Akt inhibitor VIII at 10 μM. The nuclear and cytoplasmic extracts of MM cells were harvested, and the protein levels of Sp1 were analyzed by Western blotting. The nuclear protein p84 was used as a protein loading control. **B.** RPMI8226, INA6, and MM.1S cells were cultured for 6 hours at pH7.4 or pH6.8. LY294002 and the Sp1 inhibitor terameprocol (TMP) were added at 10 μM and 50 μM, respectively, as indicated. *TRPV1* mRNA expression was analyzed by RT-PCR. *GAPDH* was used as an internal control. **C.** RPMI8226 cells were transfected with human Sp1 shRNA or control shRNA as described in Materials and Methods. These cells were cultured at pH7.4 or pH6.8. After culturing 24 and 6 hours, cell lysates and total RNA were collected. The expression of Sp1, TRPV1, and HDAC1 were analyzed by Western blotting (left) and RT-PCR (right). β-actin and *GAPDH* were used as a protein loading control and an internal control, respectively.

Sp1 has been demonstrated to be a transcription factor responsible for TRPV1 gene expression [37, 38] and constitutively overexpressed in MM cells [39–41]. Because activation of the PI3K/Akt pathway has been shown to induce nuclear localization of Sp1 in other types of cells [42–44], we next looked at nuclear localization of Sp1 in MM cells. An acidic condition induced the nuclear localization of Sp1 in MM cells, which was suppressed by addition of the PI3K inhibitor LY294002 as well as an Akt inhibitor (Figure 3A). Further, upregulation of *TRPV1* mRNA expression in MM cells in an acidic condition was suppressed by the PI3K inhibition as well as treatment with terameprocol, a competitive inhibitor of Sp1 binding to promoter regions (Figure 3B). To further confirm the role of Sp1, we examined the effects of *Sp1* gene knockdown on TRPV1 levels in MM cells. Treatment with *Sp1* shRNA effectively reduced Sp1 expression at protein levels in RPMI8226 MM cells at both pH7.4 and pH6.8 (Figure 3C). TRPV1 levels were also substantially decreased in the MM cells with the *Sp1* knockdown at pH6.8 as well as pH7.4. These results collectively suggest that an acidic condition activates the PI3K-Akt pathway in MM cells, which induces Sp1 nuclear localization and thereby TRPV1 expression to form a progressive vicious cycle between acid sensing and survival signaling.

### Histone deacetylation and gene repression in MM cells under acidic conditions

**Figure 4:**
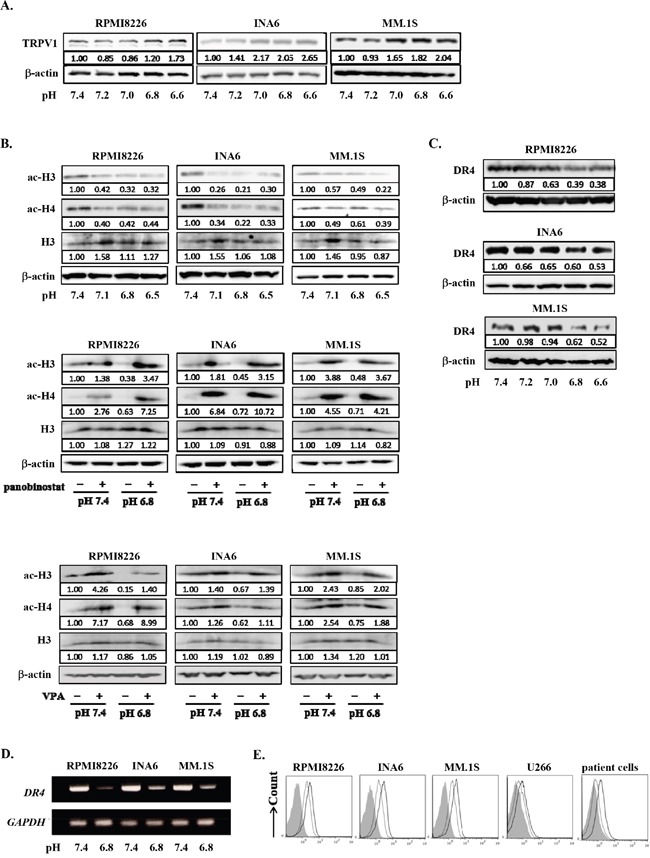
Histone acetylation and DR4 repression in MM cells under acidic conditions **A.** RPMI8226, INA6, and MM.1S cells were cultured for 24 hours at the indicated pH values. HDAC1 protein levels were analyzed by Western blotting. **B.** The cells were cultured for 24 hours at the indicated pH values in the presence or absence of the HDAC inhibitor panobinostat at 10 nM or valproate (VPA) at 100 μg/mL. The protein levels of acetylated histone H3 and histone H4 were analyzed by Western blotting. Histone H3 and β-actin were used as protein loading controls. **C.** RPMI8226, INA6, and MM.1S cells were cultured for 24 hours at the indicated pH values. The protein levels of DR4 were analyzed by Western blotting. β-actin was used as a protein loading control. **D.** The cells were cultured for 6 hours at pH7.4 or pH6.8. DR4 mRNA expression was analyzed by RT-PCR. GAPDH was used as an internal control. **E.** RPMI8226, INA6, MM.1S, and U266, as well as primary MM cells were cultured for 24 hours at pH7.4 or pH 6.8. The surface expression of DR4 was analyzed by flow cytometry. Solid and dotted lines show the expression levels of DR4 of the cells cultured at pH7.4 and pH6.8, respectively. Background staining with control IgG was shown in gray.

Sp1 is also known as a transcription factor of *HDAC1* gene [40, 45]. Besides TRPV1, HDAC1 expression was upregulated in RPMI8266 cells in an acidic condition; Sp1 knockdown abrogated their upregulation of HDAC1 expression at protein as well as mRNA levels (Figure 3C). Therefore, we next investigated the roles of acidic conditions in HDAC activity in MM cells. HDAC1 protein levels were upregulated in MM cells cultured as pH values were lowed in their culture media (Figure 4A). Conversely, acetylation of histone H3 and histone H4 was reduced in MM cells as ambient pH values were lowered (Figure 4B). The suppression of acetylation of histone H3 and histone H4 was substantially restored upon treatment with the pan-HDAC inhibitor panobinostat or the HDAC class I-specific HDAC inhibitor valproate (Figure 4B). These results suggest induction of histone deacetylation and thereby epigenetic alteration of gene expression in MM cells under acidic conditions.

Because HDAC-mediated gene repression is suggested in MM cells under acidic conditions, we examined a gene expression profile of INA6 MM cells under acidic conditions using a cDNA microarray. Because INA6 MM cells expressed TRPV1 but not other TRPV family members, TRPV2-5 (Figure 2B), we chose INA6 MM cells to better understand TRPV1-mediated acid sensing and a gene expression profile in an acidic condition. Out of 30592 genes analyzed, 2037 genes were downregulated at pH6.8 to levels less than half of those at pH7.4 in INA6 cells. Treatment with the HDAC inhibitor valproate restored the expression of 1533 genes down-regulated at pH6.8, including 962 cellular process-related genes, 810 organelle-related genes, 707 metabolic process-related genes, 483 binding-related genes, 310 membrane-enclosed lumen-related genes, and 93 catalytic activity-related genes, indicating the important role of histone deacetylation in gene repression in MM cells under acidic conditions. All raw data is available on the NCBI Gene Expression Omnibus (http://www.ncbi.nlm.nih.gov/geo/query/acc.cgi?acc=GSE52611).

Death receptor 4 (DR4) is a cognate receptor for TNF-related apoptosis-inducing ligand (TRAIL) responsible for induction of apoptosis by TRAIL. We have been studied on the regulatory mechanism of DR4 expression in MM cells to develop the strategy to enhance cytotoxic effects of TRAIL agonists as we previously reported [46]. Therefore, we are interested in the change of DR4 expression in MM cells in response to acid, and selected *DR4* as a representative gene from many differentially expressed genes in the gene expression profile in an acidic condition. According to the above gene expression profile in INA6 MM cells, a *DR4* gene was confirmed to be clearly repressed in MM cells under an acidic condition in an HDAC-dependent manner. DR4 protein levels were also found to be decreased in RPMI8226, INA6, and MM.1S cells cultured for 24 hours at acidic conditions in a pH-dependent manner (Figure 4C). Further, *DR4* mRNA expression and surface levels of DR4 were also decreased at pH6.8 (Figures 4D and Figure 4E, respectively). Therefore, *DR4* is regarded as a representative gene repressed in MM cells in acidic conditions.

### DR4 expression was suppressed in MM cells under acidic conditions in an HDAC-dependent manner

**Figure 5:**
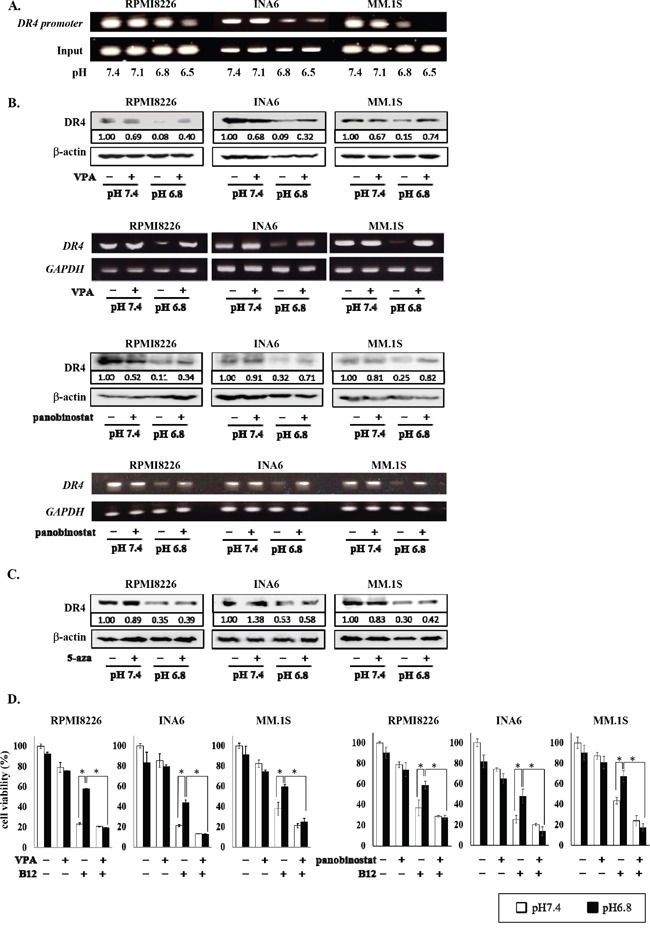
Restoration of DR4 expression in MM cells by HDAC inhibition under acidic conditions **A.** RPMI8226, INA6, and MM.1S cells were cultured at the indicated pH values for 6 hours. The acetylation of H3K9 was analyzed by the ChIP assay with the anti-acetylated H3K9 antibody as described in the Materials and Methods. Immunoprecipitated and total input DNA fractions were subject to analysis for the DR4 promoter by RT-PCR. **B.** RPMI8226, INA6, and MM.1S cells were cultured at pH7.4 or pH6.8 in the presence or absence of valproate (VPA) at 100 μg/mL or panobinostat at 10 nM. After culturing 24 and 6 hours, cell lysates and total RNA were collected, respectively. The Expression of DR4 were analyzed by Western blotting (upper) and RT-PCR (lower). β-actin and GAPDH were used as a protein loading control and an internal control, respectively. **C.** RPMI8226, INA6, and MM.1S cells were cultured for 24 hours at pH7.4 or pH6.8 with or without 5-azacitizine (5-aza) at 2.5 μM. Protein levels of DR4 were analyzed by Western blotting. β-actin was used as a protein loading control. **D.** RPMI8226, INA6, and MM.1S cells were cultured for 24 hours at pH7.4 or pH6.8 with or without the anti-DR4 agonistic antibody R1-B12 (B12) at 20 μg/mL. Valproate (VPA) at 100 μg/mL or panobinostat at 10 nM was added as indicated. Cell viability was determined by the WST-8 assay. White and black bars represented the condition of pH7.4 and pH6.8, respectively. Results are expressed as % change from the baseline with the mean +/− SD. *, P <0.01.

We next investigated the precise mechanism of epigenetic regulation of gene expression in MM cells under acidic conditions, using *DR4* as a representative gene repressed in an acidic condition in an HDAC-dependent manner. To further confirm *DR4* gene repression by histone deacetylation, we analyzed the acetylation status of histone H3K9 in a *DR4* promoter by a ChIP assay because deacetylation of the promoter region is known to suppress *DR4* gene expression. The acetylation of histone H3K9 in a *DR4* promoter was markedly reduced in MM cells under acidic conditions in a pH-dependent manner (Figure 5A).

Treatment with the HDAC inhibitors valproate as well as panobinostat restored DR4 expression suppressed at pH6.8 at protein and mRNA levels in RPMI8226, INA6, and MM.1S cells, whereas valproate and panobinostat marginally affected DR4 expression in these cells at pH7.4 (Figure 5B). However, treatment with the DNA methylation inhibitor 5-azacitidine showed only marginal effects (Figure 5C). Cross-linking of DR4 with the anti-DR4 agonistic antibody R1-B12 potently induced death in DR4-expressing RPMI8226, INA6, and MM.1S cells at pH7.4; however, the cytotoxic effects of R1-B12 were blunted at pH6.8 (Figure 5D). Because the cytotoxic effects of the anti-DR4 agonistic antibody largely depend on the surface levels of DR4 [46–48], the attenuation of R1-B12-induced MM cell death at pH6.8 appeared to be due to the down-regulation of DR4 expression in MM cells. However, HDAC inhibition by valproate as well as panobinostat restored the anti-MM effects of R1-B12 under acidic conditions (Figure 5D). These results suggest the HDAC-mediated repression of *DR4* gene in MM cells in an acidic environment blunts the efficacy of DR4-mediated immunotherapy against MM cells, which can be restored by HDAC inhibition.

### Inhibition of PI3K-Akt-Sp1 pathway restored DR4 expression down-regulated in MM cells in an acidic condition

**Figure 6:**
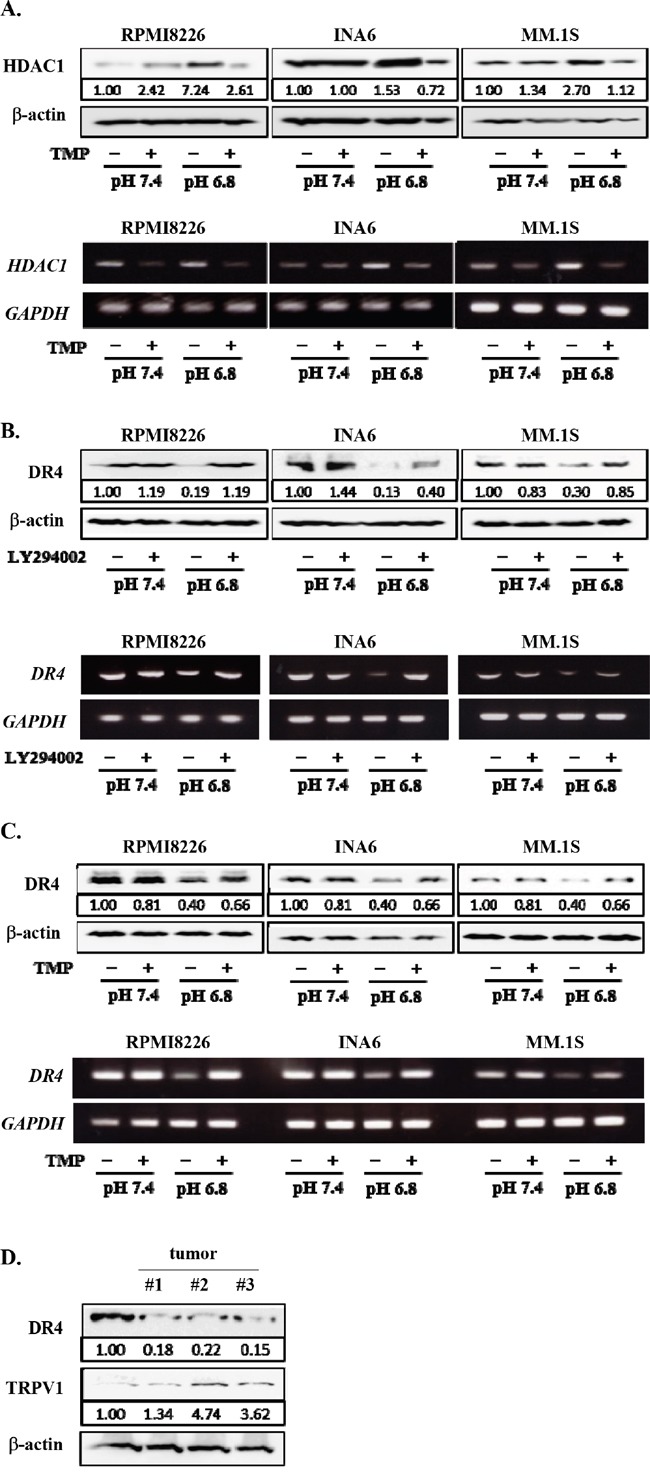
Restoration of DR4 expression in MM cells by inhibition of PI3K-Akt-Sp1 pathway under acidic conditions RPMI8226, INA6, and MM.1S cells were cultured for 6 hours at pH7.4 or pH6.8 in the presence or absence of terameprocol (TMP) at 50mM. **A.** After culturing for 24 hours, the protein levels of HDAC1 were analyzed by Western blotting. β-actin was used as a protein loading control. After culturing for 6 hours, *HDAC1* mRNA expression was analyzed by RT-PCR. GAPDH was used as an internal control. **B.** After culturing for 24 hours, DR4 protein levels were analyzed by Western blotting. β-actin was used as a protein loading control. After culturing for 6 hours, *DR4* mRNA expression was analyzed by RT-PCR. *GAPDH* was used as an internal control. **C.** The cells were cultured at pH7.4 or pH6.8 in the presence or absence of LY294002 at 10μM. After culturing 24 and 6 hours, cell lysates and total RNA were collected, respectively. The expression of DR4 were analyzed by Western blotting (upper) and RT-PCR (lower). β-actin and *GAPDH* were used as a protein loading control and an internal control, respectively. **D.** The protein levels of DR4 and TRPV1 were analyzed by Western blotting in RPMI8226 cells cultured at pH7.4 and subcutaneous tumors of RPMI8226 cells in 3 mouse MM models.

Because HDAC1 is a target gene of Sp1, and because the PI3K-Akt-Sp1 pathway was activated in MM cells in acidic conditions (Figure 3), we next investigated the role of the PI3K-Akt-Sp1 pathway in DR4 repression in MM cells in acidic conditions. Blockade of transcriptional activity of Sp1 by terameprocol as well as *Sp1* gene knockdown with *Sp1* shRNA were able to substantially suppress *HDAC1* expression in MM cells at pH7.4 and abolish upregulation of its expression at pH6.8 (Figures 3C and 6A). In addition, treatment with the Sp1 inhibitor terameprocol restored DR4 expression in MM cells suppressed at pH6.8 (Figure 6B). Consistent with the observation that the nuclear localization of Sp1 was enhanced through activation of the PI3K-Akt pathway in MM cells under acidic conditions (Figure 3A), the PI3K inhibitor LY294002 restored DR4 expression in MM cells suppressed at pH6.8 at protein as well as mRNA levels (Figure 6C), suggesting that DR4 repression in MM cells under acidic conditions is largely mediated by activation of the PI3K-Akt-Sp1 pathway. Because DR4 repression was ameliorated by HDAC inhibition in MM cells under acidic conditions (Figure 5B), these results suggest that acidic conditions activate the PI3K-Akt-Sp1-HDAC1 pathway to cause HDAC-mediated DR4 repression in MM cells.

We finally looked at the expression of DR4 and the pH sensor TRPV1 in MM tumors in mouse models with subcutaneous inoculation of RPMI8226 cells. The average pH value was found to be 6.86 inside tumors. DR4 protein levels were decreased in the *in vivo* tumors compared with RPMI8226 cells cultured at pH7.4, whereas TRPV1 was upregulated in these tumors (Figure 6D). Therefore, MM cells may enhance acid sensing while reducing DR4 expression to mitigate DR4-mediated apoptotic insults in acidic tumorous lesions *in vivo*.

## DISCUSSION

Cancer cells acidify their surrounding milieu while they grow; extracellular pH values have been reported to be 6.2–6.8 in cancer lesions compared to pH 7.2–7.4 in normal tissues [14–18]. As expected, we found the actual pH values were around 6.8 inside subcutaneous MM tumors in mouse models. MM cells stimulate OC formation and activation in the bone marrow; OCs produce a large amount of protons to form resorption pits in which pH values are generally accepted to be below 5.5 [19]. Therefore, MM bone lesions appear to serve an acidic milieu to MM cells where MM cells preferentially reside and grow. We expected pH values in acidic bone lesions in patients with MM are similar to those observed in subcutaneous MM tumors in mouse models (around 6.8) or more acidic at least in close vicinity to OCs in MM bone lesions, although there has been no information about actual pH values in bone lesions in patients with MM because of difficulty in measuring actual pH values in the bone marrow. Therefore, we looked at the effects of pH 6.8 as a representative pH value in MM tumors in the present study.

Because TRPV1 is among major pH sensors expressed in cancer cells [30–33], we first examined the effects of an antagonist and agonist of TRPV1 and found the critical role of TRPV1 in acid-induced activation of the PI3K/Akt pathway in MM cells (Figure 1B and 1C). Therefore, we focused on TRPV1 in MM cells. The present study demonstrates that the PI3K-Akt pathway is activated in MM cells under acidic conditions, which further upregulates the expression of TRPV1 to facilitate acid sensing, thereby forming a positive feedback loop between acid sensing and activation of the PI3K-Akt survival signaling. The activation of the PI3K-Akt pathway in MM cells in acidic conditions facilitated the nuclear localization of the transcription factor Sp1 responsible for *TRPV1* gene expression, which appears to be one of the mechanisms of the acid-induced upregulation of TRPV1 in MM cells. Therefore, MM cells are suggested to activate the PI3K-Akt survival pathway in response to acid to adapt to and survive in a harsh acidic tumor microenvironment.

We also found that histone H3 and histone H4 were deacetylated in MM cells in acidic conditions, while repressing a wide variety of genes. This was partly due to upregulation of HDAC1 expression in MM cells through the acid-induced nuclear localization of Sp1, which also acts as a transcription factor for *HDAC1* gene. Indeed, valproate, a HDAC inhibitor rather specific to class1 HDACs including HDAC1, restored the expression of substantial numbers of genes which were suppressed in INA6 MM cells at pH6.8, suggesting a significant contribution of gene repression in MM cells in acidic conditions by class1 HDACs.

*DR4* was found to be one of such genes repressed in a HDAC-dependent manner in an acidic condition. We further dissected the mechanism of HDAC-mediated gene repression in MM cells in acidic conditions, using *DR4*. DR4 expression was curtailed (Figures 4C, 4D and 4E), and histone H3K9 in a *DR4* gene promoter was deacetylated in MM cells (Figure 5A) in acidic conditions. However, inhibition of HDAC as well as Sp1 or PI3K was able to restore their DR4 expression curtailed in acidic conditions (Figures 5B, 6B and 6C). Such acid-induced reduction of DR4 expression protects MM cells from DR4 agonist-induced cell death (Figures 5D), which may limit DR4 agonist-based immunotherapy against MM cells. Inhibition of HDAC activity as well as Sp1 is suggested to be able to restore MM cell susceptibility to DR4 agonists in acidic conditions in MM bone lesions.

In addition to HDAC-mediated gene repression, a number of genes were upregulated in MM cells under an acidic condition. Because Sp1 transcriptionally upregulates various important genes, the roles of Sp1 in acid-induced alteration of gene expression in MM cells should be further clarified to better understand MM cell biology in acidic conditions, which may lead to development of new therapeutic options against MM.

The present study also cautions that we should take into account alteration of gene expression in cancer cells when analyzed in non-acidic culture conditions, which may cause misinterpretation of genetic profiles in cancer cells *in vivo*. We need to establish sophisticated experimental conditions to correctly reflect ambient tumor microenvironment and further clarify precise regulation of gene expression in cancer cells in pathological microenvironments surrounding them.

Based on the present study, Akt inhibitors may be able to target MM cells in acidic conditions; and HDAC inhibitors may resume the expression of various genes in MM cells in acidic conditions, which may sensitize MM cells to therapeutic agents, including an anti-DR4 agonistic antibody. Therefore, combinatory treatment with Akt inhibitors or HDAC inhibitors can be envisioned to improve the therapeutic efficacy of anti-MM agents against MM cells in acidic conditions.

## MATERIALS AND METHODS

### Reagents

The following reagents were purchased from the indicated manufacturers: rabbit polyclonal anti-human DR4 antibody, anti-human TRPV1 antibody, and anti-human HDAC1 antibody from Abcam (Cambridge, UK); mouse polyclonal anti-β-actin antibody and valproate from Sigma (St. Louis, MO); rabbit polyclonal anti-Histone H3 antibody, rabbit polyclonal anti-acetyl-histone H3 antibody and anti-acetyl-histone H4 antibody from Merck Millipore (Billerica, MA); rabbit polyclonal anti-Sp1 antibody, anti-Akt antibody, anti-phosphorylated Akt antibody, anti-PI3K antibody, anti-phosphorylated PI3K antibody, anti-STAT3 antibody, anti-phosphorylated STAT3 antibody, anti-p38 antibody, anti-phosphorylated p38 antibody, anti-p65 antibody, anti-phosphorylated p65 antibody, anti-ERK antibody, anti-phosphorylated ERK antibody, anti-JNK antibody, anti-phosphorylated JNK antibody, horseradish peroxidase (HRP)-anti-rabbit IgG and anti mouse IgG from Cell Signaling Technology (Beverly, MA); rh IGF-1 from R&D Systems (Minneapolis, MN); panobinostat from Cayman Chemical Company (Ann Arbor, MI); and 5-azacytidine and Akt inhibitor VIII from Calbiochem (Darmstadt, Germany). The human monoclonal anti-DR4 agonistic IgG antibody R1-B12 was kindly provided by Kyowa Hakko Kirin Co. Ltd. (Tokyo, Japan).

### Cells and cultures

Human MM cell lines, RPMI8226 and U266 were obtained from American Type Culture Collection (ATCC) (Rockville, MD). The MM cell lines INA6, MM.1S, were kindly provided by Dr. Renate Burger (University of Kiel, Kiel, Germany) and Dr. Steven Rosen (Northwestern University, Chicago, IL), respectively. At the beginning of this study, all the 4 cell lines were authenticated by the surface expression of CD38 and CD138 by flow cytometry and cellular morphology. MM cells were purified from bone marrow mononuclear cells from patients with MM by positive selection using CD138 microbeads and Miltenyi magnetic cell sorting system (Miltenyi Biotec, Auburn, CA) according to the manufacture's instruction. MM cells were cultured in RPMI1640 supplemented with 5% fetal bovine serum, 2 mM of L-glutamine (Sigma), 100 U/mL of penicillin G and 100 mg/mL of streptomycin (Sigma). The levels of pH in culture media were adjusted by adding lactic acid (Wako, Osaka, Japan) or sodium hydroxide (Wako). OCs were generated from peripheral blood mononuclear cells as previously reported.^13^ All procedures involving human specimens were performed with written informed consent according to the Declaration of Helsinki and using a protocol approved by the Institutional Review Board for human protection.

### Flow cytometry

Cell preparation and staining in flow cytometry were performed as described previously [13]. Briefly, approximately 10^6^ cells were incubated in 100 μL PBS with 2% human γ-globulin (Japan Blood Products Organization, Tokyo, Japan) with FITC-conjugated monoclonal antibodies on ice for 40 minutes, and then washed. Samples were analyzed by flow cytometry using EPICS-Profile (Coulter Electronics, Hialeah, FL).

### Western blot analysis

Whole cell lysate was lysed in RIPA buffer, and nuclear extract was lysed by NE-PER nuclear and cytoplasmic extraction reagent kit (Thermo Fisher Scientific, Rockford, IL, USA). These lysates were supplemented with 1 mM phenylmethylsulfonyl fluoride and protease inhibitor cocktail solution (Sigma). Cell lysates and conditioned media were electrophoresed in 10% SDS-PAGE gel and blotted onto polyvinylidene difluoride membranes (Millipore, Bedford, MA). After blocking with 5% non-fat dry milk, the membranes were incubated with primary antibodies overnight at 4°C, followed by washing and addition of a horseradish-conjugated secondary antibody for 1 hour. The protein bands were visualized with an Enhanced Chemiluminescence Plus Western Blotting Detection System (Amersham Biosciences, Piscataway, NJ).

### RT-PCR

Total RNA was extracted from cells using TRIZOL reagent (Gibco BRL, Rockville, MD). For reverse transcription-polymerase chain reaction (RT-PCR), 2 μg total RNA was reverse-transcribed with Superscript II (Gibco) in a 20μL reaction solution. One tenth of the RT-PCR products were used for subsequent PCR analysis with 24–30 cycles of 95°C for 30 seconds, 58°C for 30 seconds, and 72°C for 30 seconds. The following primers were used: DR4 forward, 5′-TGGCACACAGCAATGGGAACATAG-3′, DR4 reverse, 5′-GAAACACACCCTGTCCATGCACTT-3′, DR4 promoter forward, 5′-AGCGCAATGGCT CCATCTCGGCTC-3′, DR4 promoter reverse, 5′-AGTCACGGTCCTGCCTGCGAAGAA-3′, HDAC1 forward, 5′-AACCTGCCTATGCTGATGCTGG-3′, HDAC1 reverse, 5′-TCGTCTTCGTCCTCATCG-3′, TRPV1 forward, 5′-AACTGGACCACCTGGAACAC-3′, TRPV1 reverse 5′-GCCTGAAACTCTGCTTGACC-3′, TRPV2 forward, 5′-GTGACGGAACAGCCCACGGT-3′, TRPV2 reverse 5′-CAGTGATGCCTGGCCCTGATGG-3′, TRPV3 forward, 5′-GCTGAAGAAGCGCATCTTTGCA-3′, TRPV3 reverse 5′-TCATAGGCCTCCTCTGTGTACT-3′, TRPV4 forward, 5′-CCCGTGAGAACACCAAGTTT-3′, TRPV4 reverse 5′-TCACTCCAGGGCATTTCTTC-3′, TRPV5 forward 5′-TGATGGGTGACACACACTGG-3′, TRPV5 reverse 5′-GAAGCACTCGCAAAGGATTT-3′, GAPDH forward, 5′-AATCCCATCACCATCTTCCA-3′, GAPDH reverse, 5′-TGGACTCCACGACGTACTCA-3′.

### ChIP assay

ChIP assay was performed using a ChIP assay kit (Millipore). Cells were treated with 1% formaldehyde for 10 minutes at 37 °C. The lysates were sonicated 5 times with each time for 30 seconds, and centrifuged. The supernatants were diluted in a ChIP dilution buffer, and incubated overnight at 4 °C with anti-acetyl-H3K9 antibody. Then, 60 μL of salmon sperm DNA/protein A agarose beads-50% slurry was added, and incubated for 2 hours at 4 °C with rotation. After the incubation, the beads were pelleted, and washed with low and high salt buffers, and finally three times with LiCl buffer. The immune complexes were incubated for 4 hours at 65 °C with 500 μL of fresh elution buffer (1% SDS, 0.1 M NaHCO_3_) and 20 μL of 5 M NaCl, and eluted, followed by the addition of 2 μL of 500 mM EDTA, 1 μL of 1 M Tris and 1 μL of proteinase K. The DNA solution was extracted with a phenol/chloroform/isoamyl alcohol mixture to remove protein contaminants, and precipitated with 100% ethanol. After the precipitation step, pellets were washed with 70% ethanol, and dissolved in 20 μL distilled water.

### Cytotoxicity assay

In cytotoxic analyses with R1-B12, R1-B12 was added into cultures, followed by cross-linking with goat F(ab′)_2_ anti-human IgG Fc. After culturing for 24 hours, cell viability was determined by Cell Counting Kit-8 assay (DOJINDO, Kumamoto, Japan) according to the manufacturer's instructions. Briefly, cells were incubated in culture plates with 2-(2-methoxy-4-nitrophenyl)-3-(4-nitrophenyl)-5-(2,4-disulphophenyl)-2H-tetrazolium monosodium salt (WST-8) for 4 hours. The absorbance of each well was measured at 450 nm with a microtiter plate reader (Model 450 micro plate reader; Bio-Rad Laboratories, Hercules, CA).

### cDNA microarray

INA6 cells were cultured for 12 hours, at pH7.4 or pH6.8 in the absence or presence of valproate at 100 μg/mL. The total RNA was isolated using RNeasy Mini kit (Qiagen, Valencia, CA, USA). Cyanine-3 (Cy3) labeled cRNA was prepared from 0.13 μg RNA using the One-Color Low Input Quick Amp Labeling kit (Agilent Technologies, Santa Clara, CA) according to the manufacturer's instructions, followed by RNeasy column purification (Qiagen). Dye incorporation and cRNA yield were checked with the NanoDrop ND-1000 Spectrophotometer. 1.65 μg of Cy3-labelled cRNA (specific activity >13.0 pmol Cy3/μg cRNA) was fragmented at 60°C for 30 minutes in a reaction volume of 55 μL containing 1x Agilent fragmentation buffer and 2x Agilent blocking agent following the manufacturers instructions. On completion of the fragmentation reaction, 55 μL of 2x Agilent hybridization buffer was added to the fragmentation mixture and hybridized to Agilent Whole Human Genome Oligo Microarrays (G4845A) for 17 hours at 65°C in a rotating Agilent hybridization oven. After hybridization, microarrays were washed 1 minute at room temperature with GE wash buffer 1 (Agilent) and 1 minute with 37Â°C GE wash buffer 2 (Agilent), then dried immediately by brief centrifugation. Slides were scanned immediately after washing on the Agilent DNA Microarray Scanner (G2505C) using one color scan setting for 4x44k array slides (Scan Area 61x21.6 mm, Scan resolution 5um, Dye channel is set to Green). The scanned images were analyzed with Feature Extraction Software 10.7 (Agilent) using default parameters (protocol GE1-107_Sep09 and Grid: 026652_D_F_20110325) to obtain a background subtracted and spatially detrended Processed Signal intensities. Features flagged in Feature Extraction as Feature Non-uniform outliers were excluded. All raw data is available on the NCBI Gene Expression Omnibus (http://www.ncbi.nlm.nih.gov/geo/query/acc.cgi?acc=GSE52611).

### Short-hairpin RNA (shRNA) transduction

shRNA lentiviral transduction particles (Sigma-Aldrich) were used and shRNA transduction was performed with the MagnetoFection-ViroMag R/L (OZ Biosciences, Marseille, France) as previously described. Sp1 shRNA (NM_138473.2-5s21c1) (Sigma-Aldrich) or control shRNA (Sigma-Aldrich) were designed by MISSION^®^. To increase the transduction efficiency, 1 mL of RPMI8226 cell suspension (10^5^ cells) was mixed with 20 μL CombiMag^®^ (OZ Biosciences) and incubated for 15 minutes. Then, the cells were distributed to 96-well plates (100 μL/well) and placed for 15 minutes on a magnetic plate. To prepare lentiviral transduction particles, 1 multiplicity of infection (MOI) of lentiviral particles were added to 2 μL of ViroMag R/L beads (OZ Biosciences), and incubated for 15 minutes at room temperature. Then the virus particles/ViroMag R/L were added to each well, and the cells were incubated on the magnet plate for 60 minutes at room temperature. The culture plates were removed from the magnetic plate and the cells were cultured at 37°C with 5% CO_2_ for 24 hours. To establish stable Sp1 knockdown cells, clones were selected by treatment with puromycin (Sigma) at 5 μg/mL.

### Mouse MM model

RPMI8226 cells (5x10^6^ cells/mouse) were subcutaneously inoculated to SCID mice pretreated with anti-asyaloGM1 antibody (Wako, Osaka, Japan), as previously described [49, 50]. After enlarging subcutaneous tumors more than 2 cm in diameter, we inserted pH sensors (InLab, Mettler Toled, Columbus, OH) directly into tumors or subcutaneous regions outside tumors and measured pH values. After measuring pH, tumors were resected to prepare cell lysates.

### Statistical analysis

Statistical significance was determined by one-way analysis of variance (ANOVA) with Scheffe's post hoc tests. The minimal level of significance was *P*=0.05.

## References

[1] Pearse RN, Sordillo EM, Yaccoby S, Wong BR, Liau DF, Colman N, Michaeli J, Epstein J, Choi Y (2001). Multiple myeloma disrupts the TRANCE/osteoprotegerin cytokine axis to trigger bone destruction and promote tumor progression. Proc Natl Acad Sci U S A.

[2] Giuliani N, Bataille R, Mancini C, Lazzaretti M, Barille S (2001). Myeloma cells induce imbalance in the osteoprotegerin/osteoprotegerin ligand system in the human bone marrow environment. Blood.

[3] Abe M, Hiura K, Wilde J, Moriyama K, Hashimoto T, Ozaki S, Wakatsuki S, Kosaka M, Kido S, Inoue D, Matsumoto T (2002). Role for macrophage inflammatory protein (MIP)-1alpha and MIP-1beta in the development of osteolytic lesions in multiple myeloma. Blood.

[4] Choi SJ, Cruz JC, Craig F, Chung H, Devlin RD, Roodman GD, Alsina M (2000). Macrophage inflammatory protein 1-alpha is a potential osteoclast stimulatory factor in multiple myeloma. Blood.

[5] Tian E, Zhan F, Walker R, Rasmussen E, Ma Y, Barlogie B, Shaughnessy JD (2003). The role of the Wnt-signaling antagonist DKK1 in the development of osteolytic lesions in multiple myeloma. N Engl J Med.

[6] Oshima T, Abe M, Asano J, Hara T, Kitazoe K, Sekimoto E, Tanaka Y, Shibata H, Hashimoto T, Ozaki S, Kido S, Inoue D, Matsumoto T (2005). Myeloma cells suppress bone formation by secreting a soluble Wnt inhibitor, sFRP-2. Blood.

[7] Takeuchi K, Abe M, Hiasa M, Oda A, Amou H, Kido S, Harada T, Tanaka O, Miki H, Nakamura S, Nakano A, Kagawa K, Yata K (1371). Tgf-Beta inhibition restores terminal osteoblast differentiation to suppress myeloma growth. PLoS One.

[8] Vallet S, Mukherjee S, Vaghela N, Hideshima T, Fulciniti M, Pozzi S, Santo L, Cirstea D, Patel K, Sohani AR, Guimaraes A, Xie W, Chauhan D (1073). Activin A promotes multiple myeloma-induced osteolysis and is a promising target for myeloma bone disease. Proc Natl Acad Sci U S A.

[9] D'Souza S, del Prete D, Jin S, Sun Q, Huston AJ, Kostov FE, Sammut B, Hong CS, Anderson JL, Patrene KD, Yu S, Velu CS, Xiao G (1182). Gfi1 expressed in bone marrow stromal cells is a novel osteoblast suppressor in patients with multiple myeloma bone disease. Blood.

[10] Raje N, Roodman GD (2011). Advances in the biology and treatment of bone disease in multiple myeloma. Clin Cancer Res.

[11] Abe M (2011). Targeting the interplay between myeloma cells and the bone marrow microenvironment in myeloma. Int J Hematol.

[12] Yaccoby S (2010). Advances in the understanding of myeloma bone disease and tumour growth. Br J Haematol.

[13] Abe M, Hiura K, Wilde J, Shioyasono A, Moriyama K, Hashimoto T, Kido S, Oshima T, Shibata H, Ozaki S, Inoue D, Matsumoto T (2004). Osteoclasts enhance myeloma cell growth and survival via cell-cell contact: a vicious cycle between bone destruction and myeloma expansion. Blood.

[14] Brisson L, Reshkin SJ, Gore J, Roger S (1016). pH regulators in invadosomal functioning: proton delivery for matrix tasting. Eur J Cell Biol.

[15] Harhaji L, Popadic D, Miljkovic D, Cvetkovic I, Isakovic A, Trajkovic V (2006). Acidosis affects tumor cell survival through modulation of nitric oxide release. Free Radic Biol Med.

[16] Gerweck LE, Vijayappa S, Kozin S (2006). Tumor pH controls the *in vivo* efficacy of weak acid and base chemotherapeutics. Mol Cancer Ther.

[17] Zhang X, Lin Y, Gillies RJ (1167). Tumor pH and its measurement. J Nucl Med.

[18] Lardner A (2001). The effects of extracellular pH on immune function. J Leukoc Biol.

[19] Kato Y, Ozawa S, Miyamoto C, Maehata Y, Suzuki A, Maeda T, Baba Y (2013). Acidic extracellular microenvironment and cancer. Cancer Cell Int.

[20] Tannock IF, Rotin D (1989). Acid pH in tumors and its potential for therapeutic exploitation. Cancer Res.

[21] Izumi H, Torigoe T, Ishiguchi H, Uramoto H, Yoshida Y, Tanabe M, Ise T, Murakami T, Yoshida T, Nomoto M, Kohno K (2003). Cellular pH regulators: potentially promising molecular targets for cancer chemotherapy. Cancer Treat Rev.

[22] Rofstad EK, Mathiesen B, Kindem K, Galappathi K (2006). Acidic extracellular pH promotes experimental metastasis of human melanoma cells in athymic nude mice. Cancer Res.

[23] Zhu J, Wang M, Cao B, Hou T, Mao X (2014). Targeting the phosphatidylinositol 3-kinase/AKT pathway for the treatment of multiple myeloma. Curr Med Chem.

[24] Engelman JA (2009). Targeting PI3K signalling in cancer: opportunities, challenges and limitations. Nat Rev Cancer.

[25] Chapman MA, Lawrence MS, Keats JJ, Cibulskis K, Sougnez C, Schinzel AC, Harview CL, Brunet JP, Ahmann GJ, Adli M, Anderson KC, Ardlie KG, Auclair D (1038). Initial genome sequencing and analysis of multiple myeloma. Nature.

[26] Ihara Y, Kihara Y, Hamano F, Yanagida K, Morishita Y, Kunita A, Yamori T, Fukayama M, Aburatani H, Shimizu T, Ishii S (2010). The G protein-coupled receptor T-cell death-associated gene 8 (TDAG8) facilitates tumor development by serving as an extracellular pH sensor. Proc Natl Acad Sci U S A.

[27] Ludwig MG, Vanek M, Guerini D, Gasser JA, Jones CE, Junker U, Hofstetter H, Wolf RM, Seuwen K (2003). Proton-sensing G-protein-coupled receptors. Nature.

[28] Murakami N, Yokomizo T, Okuno T, Shimizu T (2004). G2A is a proton-sensing G-protein-coupled receptor antagonized by lysophosphatidylcholine. J Biol Chem.

[29] de la Roche J, Eberhardt MJ, Klinger AB, Stanslowsky N, Wegner F, Koppert W, Reeh PW, Lampert A, Fischer MJ, Leffler A (2013). The molecular basis for species-specific activation of human TRPA1 protein by protons involves poorly conserved residues within transmembrane domains 5 and 6. J Biol Chem.

[30] Alptekin M, Eroglu S, Tutar E, Sencan S, Geyik MA, Ulasli M, Demiryurek AT, Camci C (2015). Gene expressions of TRP channels in glioblastoma multiforme and relation with survival. Tumour Biol.

[31] Yoneda T, Hiasa M, Nagata Y, Okui T, White F (2015). Contribution of acidic extracellular microenvironment of cancer-colonized bone to bone pain. Biochim Biophys Acta.

[32] de Jong PR, Takahashi N, Harris AR, Lee J, Bertin S, Jeffries J, Jung M, Duong J, Triano AI, Niv Y, Herdman DS, Taniguchi K, Kim CW (2014). Ion channel TRPV1-dependent activation of PTP1B suppresses EGFR-associated intestinal tumorigenesis. J Clin Invest.

[33] Wu TT, Peters AA, Tan PT, Roberts-Thomson SJ, Monteith GR (2014). Consequences of activating the calcium-permeable ion channel TRPV1 in breast cancer cells with regulated TRPV1 expression. Cell Calcium.

[34] Kyle RA (1995). Prognostic factors in multiple myeloma. Stem Cells.

[35] Qiang YW, Yao L, Tosato G, Rudikoff S (2004). Insulin-like growth factor I induces migration and invasion of human multiple myeloma cells. Blood.

[36] Nakano A, Miki H, Nakamura S, Harada T, Oda A, Amou H, Fujii S, Kagawa K, Takeuchi K, Ozaki S, Matsumoto T, Abe M (2012). Up-regulation of hexokinaseII in myeloma cells: targeting myeloma cells with 3-bromopyruvate. J Bioenerg Biomembr.

[37] Chu C, Zavala K, Fahimi A, Lee J, Xue Q, Eilers H, Schumacher MA (2011). Transcription factors Sp1 and Sp4 regulate TRPV1 gene expression in rat sensory neurons. Mol Pain.

[38] Zavala K, Lee J, Chong J, Sharma M, Eilers H, Schumacher MA (2014). The anticancer antibiotic mithramycin-A inhibits TRPV1 expression in dorsal root ganglion neurons. Neurosci Lett.

[39] Fulciniti M, Amin S, Nanjappa P, Rodig S, Prabhala R, Li C, Minvielle S, Tai YT, Tassone P, Avet-Loiseau H, Hideshima T, Anderson KC, Munshi NC (2011). Significant biological role of sp1 transactivation in multiple myeloma. Clin Cancer Res.

[40] Kikuchi J, Wada T, Shimizu R, Izumi T, Akutsu M, Mitsunaga K, Noborio-Hatano K, Nobuyoshi M, Ozawa K, Kano Y, Furukawa Y (2010). Histone deacetylases are critical targets of bortezomib-induced cytotoxicity in multiple myeloma. Blood.

[41] Mao X, Zhu X, Hurren R, Ezzat S, Schimmer AD (2008). Dexamethasone increases ubiquitin transcription through an SP-1 dependent mechanism in multiple myeloma cells. Leuk Res.

[42] Amodio N, Di Martino MT, Foresta U, Leone E, Lionetti M, Leotta M, Gulla AM, Pitari MR, Conforti F, Rossi M, Agosti V, Fulciniti M, Misso G (2012). miR-29b sensitizes multiple myeloma cells to bortezomib-induced apoptosis through the activation of a feedback loop with the transcription factor Sp1. Cell Death Dis.

[43] Takao T, Asanoma K, Tsunematsu R, Kato K, Wake N (2012). The maternally expressed gene Tssc3 regulates the expression of MASH2 transcription factor in mouse trophoblast stem cells through the AKT-Sp1 signaling pathway. J Biol Chem.

[44] Chuang CW, Pan MR, Hou MF, Hung WC (2013). Cyclooxygenase-2 up-regulates CCR7 expression via AKT-mediated phosphorylation and activation of Sp1 in breast cancer cells. J Cell Physiol.

[45] Wada T, Kikuchi J, Nishimura N, Shimizu R, Kitamura T, Furukawa Y (2009). Expression levels of histone deacetylases determine the cell fate of hematopoietic progenitors. J Biol Chem.

[46] Kagawa K, Nakano A, Miki H, Oda A, Amou H, Takeuchi K, Nakamura S, Harada T, Fujii S, Yata K, Ozaki S, Matsumoto T, Abe M (1371). Inhibition of TACE activity enhances the susceptibility of myeloma cells to TRAIL. PLoS One.

[47] Fulda S, Debatin KM (2005). Resveratrol-mediated sensitisation to TRAIL-induced apoptosis depends on death receptor and mitochondrial signalling. Eur J Cancer.

[48] Uno K, Inukai T, Kayagaki N, Goi K, Sato H, Nemoto A, Takahashi K, Kagami K, Yamaguchi N, Yagita H, Okumura K, Koyama-Okazaki T, Suzuki T (2003). TNF-related apoptosis-inducing ligand (TRAIL) frequently induces apoptosis in Philadelphia chromosome-positive leukemia cells. Blood.

[49] Miki H, Ozaki S, Nakamura S, Oda A, Amou H, Ikegame A, Watanabe K, Hiasa M, Cui Q, Harada T, Fujii S, Nakano A, Kagawa K (2013). KRN5500, a spicamycin derivative, exerts anti-myeloma effects through impairing both myeloma cells and osteoclasts. Br J Haematol.

[50] Ikegame A, Ozaki S, Tsuji D, Harada T, Fujii S, Nakamura S, Miki H, Nakano A, Kagawa K, Takeuchi K, Abe M, Watanabe K, Hiasa M (2012). Small molecule antibody targeting HLA class I inhibits myeloma cancer stem cells by repressing pluripotency-associated transcription factors. Leukemia.

